# Change in serum KL-6 level from baseline is useful for predicting life-threatening EGFR-TKIs induced interstitial lung disease

**DOI:** 10.1186/1465-9921-12-97

**Published:** 2011-07-26

**Authors:** Shigeo Kawase, Noboru Hattori, Nobuhisa Ishikawa, Yasushi Horimasu, Kazunori Fujitaka, Osamu Furonaka, Takeshi Isobe, Seigo Miyoshi, Hironobu Hamada, Takashi Yamane, Akihito Yokoyama, Nobuoki Kohno

**Affiliations:** 1 Department of Molecular and Internal Medicine, Graduate School of Biomedical Sciences, Hiroshima University, 1-2-3 Kasumi, Minami-ku, Hiroshima 734-8551, Japan; 2 Department of Respiratory Medicine, Onomichi General Hospital, 7-19 Kohama, Onomichi, Hiroshima 722-8508, Japan; 3 Department of Clinical Oncology and Respiratory Medicine, Shimane University, 89-1, Enya-cho, Izumo, Shimane 693-8501, Japan; 4 Department of Integrated Medicine and Informatics, Ehime University Graduate School of Medicine, Toon, Ehime 791-0295, Japan; 5 Department of Health and Sports Medical Sciences, Graduate School of Health Sciences, Hiroshima University, Hiroshima, Japan; 6 Department of Hematology and Respiratory Medicine, Kochi Medical School, Kochi University, Nankoku, Kochi 783-8505, Japan

**Keywords:** Lung cancer, KL-6, EGFR-TKI, interstitial lung disease

## Abstract

**Background:**

A high incidence of interstitial lung disease (ILD) has been reported in patients with advanced non-small cell lung cancer (NSCLC) treated with epidermal growth factor receptor-tyrosine kinase inhibitors (EGFR-TKIs), particularly in Japanese populations. A previous report from our laboratory demonstrated that KL-6 was a useful serum biomarker to assess the severity of drug-induced pneumonitis. Based on these observations, this study was conducted to evaluate the risk factors of EGFR-TKIs induced ILD and the usefulness of monitoring serum KL-6 levels in patients who developed EGFR-TKIs induced ILD in a large multi-institutional setting.

**Methods:**

We retrospectively reviewed clinical records and radiographies of 341 patients with advanced NSCLCs who were treated with EGFR-TKIs, and analyzed risk factors for the development of EGFR-TKIs induced ILD. Changes of circulating levels of KL-6 were also evaluated in the patients who developed EGFR-TKIs induced ILD.

**Results:**

Among the 341 patients included in this study, 20 (5.9%) developed EGFR-TKIs induced ILD, and 9 (2.6%) died from ILD. Univariate analyses revealed that only preexisting pulmonary fibrosis was a significant risk factor for the development of EGFR-TKIs induced ILD (*p *= 0.003). Absolute levels of circulating KL-6 at neither baseline nor the onset of ILD could discriminate between life-threatening and non-life threatening EGFR-TKIs induced ILDs. However, we found that the ratios of serum KL-6 levels just after the onset of EGFR-TKIs induced ILD to those at baseline could quite precisely distinguish survivors from non-survivors (*p *= 0.006) as well as acute interstitial pneumonia (AIP) pattern from non-AIP pattern (*p *= 0.005).

**Conclusions:**

The results of this study strongly support the potential of KL-6 as a diagnostic biomarker for life-threatening EGFR-TKIs induced ILD. Monitoring of KL-6 is also useful to evaluate the progression and severity of EGFR-TKIs induced ILD.

## Background

Gefitinib (ZD1839, Iressa; AstraZeneca) and erlotinib (Tarceva, OSI-774; OSI Pharmaceuticals) are orally active epidermal growth factor receptor tyrosine kinase inhibitors (EGFR-TKIs) used for the treatment of non-small cell lung cancer (NSCLC) patients [1]. EGFR-TKIs sometimes cause drastic tumor regression in specific subgroups of patients with advanced NSCLC, including women, non-smokers, patients with lung adenocarcinoma (ADC) histology, patients of Asian origin and patients with *EGFR *mutations [2-6]. On the other hand, treatment with EGFR-TKIs is associated with serious side effects, such as life-threatening drug-induced interstitial lung disease (ILD), particularly in Japanese populations [7-13]. These previous studies have reported that male gender, smoking history, poor performance status (PS), and preexisting ILD are risk factors for developing EGFR-TKIs induced ILD, however, we questioned whether each of these should be equally considered for the risk-benefit assessment to use EGFR-TKIs for the treatment of NSCLCs in a practical clinical setting. In addition, we also wondered whether we can assess the severity of EGFR-TKIs induced ILD when it develops during EGFR-TKIs treatment.

KL-6 is a mucin-like glycoprotein with a molecular weight of 200kd and has been classified as human MUC1 mucin [14-17]. Previous studies have demonstrated that serum levels of KL-6 are elevated in a variety of ILDs, such as idiopathic pulmonary fibrosis (IPF), collagen vascular disease associated interstitial pneumonitis, radiation pneumonitis, pulmonary sarcoidosis [18-26]. Furthermore, our laboratory has also demonstrated that absolute levels of KL-6 at the onset of drug-induced ILD can predict the clinical outcomes [27]. Although our previous studies have suggested the usefulness of KL-6 as a tumor marker [28,29] and a predictor of survival in NSCLC patients treated with EGFR-TKIs [30], significance of circulating KL-6 level as a detector of EGFR-TKIs induced ILD or a predictor of clinical outcome in patients with EGFR-TKIs induced ILD has not been determined yet.

In the cohort of the present study, to obtain more information on risk factors for developing EGFR-TKIs induced ILD, the characteristics of NSCLC patients who developed ILD during EGFR-TKIs treatment were analyzed. In addition, to evaluate whether monitoring serum KL-6 levels in NSCLC patients during the treatment is useful to detect the development of EGFR-TKIs induced ILD or predict the clinical outcome of EGFR-TKIs induced ILD, circulating KL-6 levels were measured in NSCLC patients included in the cohort before and during EGFR-TKIs treatment.

## Methods

### Study subjects

Between August 2002 and August 2010, 341 advanced NSCLC patients treated with gefitinib (250 mg/day) or erlotinib (150 mg/day) at Hiroshima University Hospital (Hiroshima, Japan), Ehime University Hospital (Ehime, Japan), Shimane University Hospital (Shimane, Japan), Kochi University Hospital (Kochi, Japan) and Onomichi General Hospital (Hiroshima, Japan) were consecutively enrolled in the study. The disease staging was carried out using computed tomography (CT) scan of the chest and abdomen, bone scintigraphy or F-18 fluorodeoxyglucose positron emission tomography (FDG-PET/CT), and magnetic resonance imaging (MRI) of the head. To obtain information on both the response of tumor to EGFR-TKIs treatment and the occurrence of EGFR-TKIs induced ILD, chest radiography and/or CT scans were performed at least once a month at each institution, and the patients were followed-up until 12 weeks after the administration of EGFR-TKIs. Informed consent was obtained from all patients. This study complied with the Declaration of Helsinki, and was approved by the individual institutional Ethical Committees.

### Diagnosis of preexisting pulmonary disorder and EGFR-TKIs induced ILD

The presence of preexisting pulmonary fibrosis was determined according to the diagnostic criteria set by the ATS/ERS on the basis of clinical characteristic and/or chest CT findings, and the types of preexisting pulmonary fibrosis were classified into idiopathic pulmonary fibrosis (IPF) pattern and non-IPF pattern [31-33]. In addition, the presence of preexisting pulmonary emphysema was determined by chest CT findings that show low attenuation areas occupying more than 25% of the entire lung field in at least one slice [34]. The diagnosis of EGFR-TKIs induced ILD was made using the diagnostic algorithm described elsewhere [11,35]. We defined EGFR-TKIs induced ILD as diffuse pulmonary infiltrates newly developed during EGFR-TKIs treatment with lack of evidence for alternative diseases such as infection, tumor progression, heart failure and pulmonary embolism. When the occurrence of EGFR-TKIs induced ILDs was suspected, chest CT scans were performed, levels of brain natriuretic peptide (BNP) and D-dimer in blood were measured, the sputum culture, blood culture, urine antigen test for *Legionella pneumophila *and *Streptococcus pneumoniae*, cytomegalovirus antigen test, and polymerase chain reaction test for *Pneumocystis jiroveci *were conducted. When possible, bronchoalveolar lavage or lung biopsy was carried out. Tumor progression was carefully excluded on the basis of the clinical information including chest CT findings, physical examinations, and tumor markers. The final diagnosis of EGFR-TKIs induced ILD was made by the consensus of at least two independent pulmonologists. We collected the clinical information of all 341 patients, such as patient age, sex, histologic type, disease stage, performance status, prior chemotherapy and thoracic radiation therapy, preexisting pulmonary fibrosis, preexisting pulmonary emphysema, *EGFR *mutation status, types of EGFR-TKIs, duration of EGFR-TKIs treatment and laboratory data.

### Subclassification of EGFR-TKIs induced ILD

The chest radiography and CT of the patients who developed EGFR-TKIs induced ILD were reviewed separately by two independent observers who were not aware of the patients' profiles, and were categorized into four patterns as previously described [27,36]: (1) acute interstitial pneumonia (AIP) pattern characterized by extensive bilateral ground glass attenuation or airspace consolidations with traction bronchiectasis, (2) chronic interstitial pneumonia (CIP) pattern characterized by fibrosis and/or consolidation, (3) cryptogenic organizing pneumonia/eosinophilic pneumonia (COP/EP) pattern showing peribronchial or subpleural consolidation without fibrosis, and (4) hypersensitivity pneumonitis (HP) pattern with diffuse ground glass opacities without fibrosis.

### *EGFR *mutation status

In 148 out of 341 NSCLC patients included in the study, *EGFR *mutation statuses were assessed using paraffin-embedded biopsy samples or surgically resected tumor tissues. To evaluate *EGFR *mutations, the peptide nucleic acid-locked nucleic acid polymerase chain reaction (PNA-LNA PCR) clamp test that can detect G719C, G719S, G719A, L858R, L861Q, T790M and 7 different exon 19 deletions [37] was used.

### Electrochemiluminescence immunoassay (ECLIA) to determine circulating levels of KL-6

At least one serum sample was obtained before the EGFR-TKIs treatment from each patient included in the study. From 15 out of 20 patients who developed EGFR-TKIs induced ILD, a total of 2-5 serum samples per patient were also collected weekly after the occurrence of EGFR-TKIs induced ILD, and stored at -80°C. Serum KL-6 levels were measured by sandwich-type electrochemiluminescence immunoassay (ECLIA) using a Picolumi 8220 Analyzer (Eidia, Tokyo, Japan), as previously described [29,30].

### Statistical analysis

The data were analyzed with a statistical software package (JMP, version 7.0.1; SAS Institute Inc.; Cary, North Carolina) and *p *< 0.05 indicated a significant difference. Data are shown as the mean ± SEM. Differences between patients with and without preexisting pulmonary fibrosis, survivors and non-survivors, and patients with AIP pattern and the other patterns of EGFR-TKIs induced ILD were analyzed using the Mann-Whitney U-test. We analyzed differences between patients with preexisting pulmonary fibrosis who developed EGFR-TKIs induced ILD or not using the Fisher's exact test. In order to test differences among the variables evaluated prior to and at the diagnosis of EGFR-TKIs induced ILD, Wilcoxon test was used. The risk factors associated with EGFR-TKIs induced ILD were evaluated using multiple logistic regression analysis. The criterion for removing a variable was the likelihood ratio statistic, which was based on the maximum partial likelihood estimate (default *p*-value of 0.05 for removal from the model).

## Results

### Characteristics of patients

**Figure 1:**
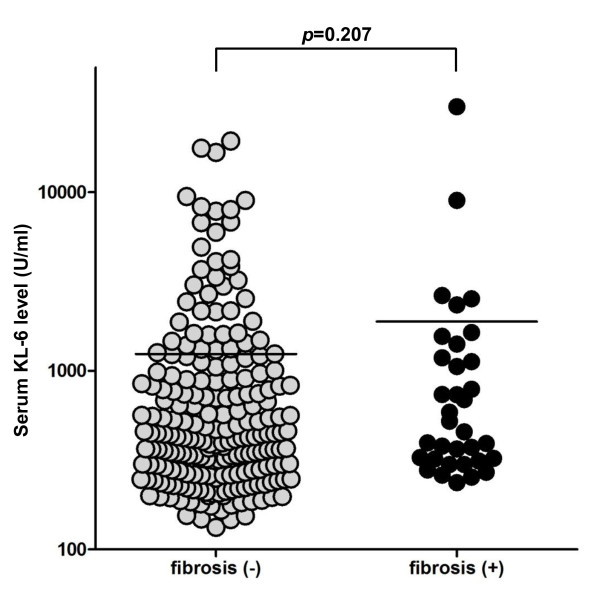
**Absolute serum levels of KL-6 at baseline in patients with and without preexisting pulmonary fibrosis**. Each point represents the absolute serum KL-6 level at baseline in patients with and without preexisting pulmonary fibrosis. There was no significant difference between the two groups (*p *= 0.207, Mann-Whitney U-test).

Table 1 shows the characteristics of the 341 patients enrolled in this study. All patients were Japanese. The ages of the patients ranged from 30 to 87 years (mean age 65.2 ± 0.6 SEM). Of the patients, 167 (49.0%) were female, 296 (86.8%) had adenocarcinomas (ADCs), 171 (50.1%) were never smokers, and 200 (58.7%) were in good performance status (PS = 0, 1). Forty-seven (13.8%) patients received thoracic radiations prior to EGFR-TKIs treatment, and preexisting ILDs were identified in 48 (14.1%) patients. Twenty-six (55.3%) out of the 47 patients who underwent radiation therapy had preexisting pulmonary fibrosis. Preexisting pulmonary emphysema was identified in 82 (24.0%) patients. PNA-LNA-PCR clamp tests to detect *EGFR *mutations could be performed in 148 (43.4%) patients, and in 91 patients, *EGFR *mutations were detected: L858R mutation in 38 patients, G719S mutation in 2 patients, exon 19 deletions in 45 patients, and other types of mutations in 6 patients. Figure 1 shows the absolute serum KL-6 levels at the baseline according to the presence of preexisting pulmonary fibrosis. The absolute serum KL-6 levels at the baseline showed no significant difference between patients with and without preexisting pulmonary fibrosis (Mann-Whitney U-test; *p *= 0.207). Table 2 shows the characteristics of the 48 patients who had preexisting pulmonary fibrosis. Eight (16.7%) out of the 48 patients with preexisting pulmonary fibrosis developed EGFR-TKIs induced ILD. Statistical analyses were made to see the association between the patients' characteristic and the development of EGFR-TKIs induced ILD among these patients (Table 2). In the patients who had preexisting pulmonary fibrosis, thoracic radiation prior to EGFR-TKIs treatment was not associated with the development of EGFR-TKIs induced ILD, however, there was a weak but statistically significant association between the development of EGFR-TKIs induced ILD and *EGFR *mutation status (*p *= 0.0498).

**Table 1:** Patients' characteristics of 341 patients treated with EGFR-TKIs

Characteristics	No. of patients	% patients
Total	341	100
Age (years)		
Mean (± SEM)	65.2(± 0.6)	
< 60	102	29.9
≥ 60	239	70.1
Sex		
Female	167	49.0
Male	174	51.0
Histologic type		
Adenocarcinoma	296	86.8
Squamous cell carcinoma	34	10.0
Others	11	3.2
Smoking history		
Current	60	17.6
Former	110	32.3
Never	171	50.1
Disease stage		
IV	206	60.4
IIIB	54	15.8
I-IIIA	18	5.3
Recurrence after surgery	63	18.5
Performance status		
≥ 2	141	41.3
0-1	200	58.7
No. of prior chemotherapy regimens		
≥ 2	118	34.6
0-1	223	65.4
Prior thoracic radiotherapy		
Yes	47	13.8
No	294	86.2
Preexisting pulmonary fibrosis		
Yes	48	14.1
No	293	85.9
Preexisting pulmonary emphysema		
Yes	82	24.0
No	259	76.0
*EGFR *mutation status		
Wild type	57	16.7
Mutant	91	26.4
Not evaluated	193	56.9
Types of EGFR-TKI		
Gefitinib	302	88.6
Erlotinib	39	11.4

**Table 2:** Patients' Characteristics of 48 patients with preexisting pulmonary fibrosis

Characteristics	Total	EGFR-TKIs induced ILD (+)	EGFR-TKIs induced ILD (-)	p-value
Total	48	8	40	
Age (years)				
Mean (± SEM)		67.5(± 3.6)	66.2(± 1.8)	
< 60	12	2	10	1.000
≥ 60	36	6	30	
Sex				
Female	11	3	8	0.361
Male	37	5	32	
Histologic type				
Adenocarcinoma	37	5	32	0.361
Squamous cell carcinoma/Others	11	3	8	
Smoking history				
Current/Former	40	5	35	0.116
Never	8	3	5	
Disease stage				
IV	24	3	21	0.701
I-IIIB/Recurrence after surgery	11	5	19	
Performance status				
≥ 2	26	5	21	0.710
0-1	22	3	19	
No. of prior chemotherapy regimens				
≥ 2	20	1	19	0.116
0-1	28	7	21	
Prior thoracic radiotherapy				
Yes	10	0	10	0.177
No	38	8	30	
Pattern of preexisting pulmonary fibrosis				
IPF pattern	3	1	2	0.429
Non-IPF pattern	45	7	38	
Preexisting pulmonary emphysema				
Yes	24	3	21	0.701
No	24	5	19	
*EGFR *mutation status				
Wild type	9	5	4	0.0498*
Mutant	11	1	10	
(Not evaluated)	(28)	(2)	(26)	
Types of EGFR-TKI				
Gefitinib	40	5	35	0.116
Erlotinib	8	3	5	

*p < 0.05 (Fisher's exact test)

### Incidence and characteristics of patients with EGFR-TKIs induced ILD

**Figure 2:**
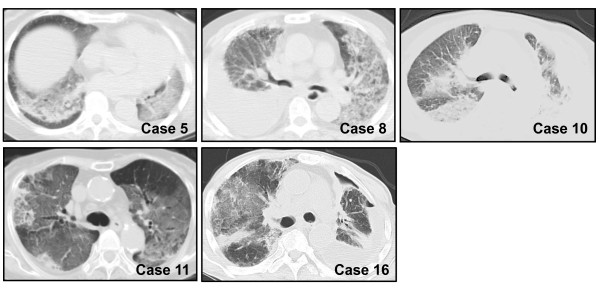
**Chest CT images of five patients who developed EGFR-TKI induced acute interstitial pneumonia (AIP)**. Representative chest CT images of the five patients who developed AIP pattern of EGFR-TKIs induced ILD are shown. Each case number corresponds to the patient's number listed in Table 3.

Among the 341 patients included in this study, 20 (5.9%) developed EGFR-TKIs induced ILD, and 9 (2.6%) died from ILD. Table 3 shows the characteristics and clinical course of these 20 patients. All the patients had acute onset or exacerbation of respiratory symptoms. The median interval from the administration of EGFR-TKI to the occurrence of EGFR-TKIs induced ILD was 19 days (range 5-51 days). The subclassifications of EGFR-TKIs induced ILD categorized by the findings of chest CT scans in these 20 patients were as follows: AIP pattern in 5 patients, COP/EP pattern in 9 patients, and HP pattern in 6 patients. The CT images of 5 patients who demonstrated AIP pattern are shown in Figure 2. When the occurrence of EGFR-TKIs induced ILD was suspected, the administration of EGFR-TKI was immediately stopped and high dose methylprednisolone (1,000 mg daily for 3 days) therapy was started. All of the 5 patients with AIP patterns were refractory to the treatment and eventually died, whereas 7 of 9 patients with COP/EP pattern and 4 of 6 patients with HP pattern showed immediate response to the treatment. Postmortem examinations were performed in 3 patients (patient No. 5, 8 and 11) and diffuse alveolar damage (DAD) was detected histologically in all of them. In addition, the presence of preexisting pulmonary fibrosis was suspected in 2 of the 3 patients. Neither infection nor lymphangitic spread of cancer cells was pointed out in any of them.

**Table 3:** Characteristics of 20 patients with EGFR-TKIs induced ILD

No	Age	Sex	Histological type	Smoking history	Stage	PS	Prior CT	Prior RT	Preexisting fibrosis	Preexisting emphysema	*EGFR *mutation	Type of EGFR-TKI	Length of EGFR-TKI	CT findings	Prognosis
1	68	M	SCC	Ex	IIIB	1	1	No	No	Yes	N.E.	Gefitinib	11	COP/EP	Alive
2	80	M	SCC	Never	Rec	2	1	No	Yes	Yes	Wild	Gefitinib	17	COP/EP	Alive
3	70	F	SCC	Never	IIIA	1	1	Yes	No	No	N.E.	Gefitinib	24	HP	Alive
4	60	M	ADC	Never	IIIB	1	1	No	No	No	N.E.	Gefitinib	35	COP/EP	Alive
5	68	F	ADC	Ex	Rec	1	2	No	No	No	Wild	Gefitinib	16	AIP	Dead
6	60	M	ADC	Current	IIIB	1	2	No	No	Yes	N.E.	Gefitinib	26	COP/EP	Alive
7	57	M	ADC	Never	Rec	0	3	No	No	No	L858R	Gefitinib	51	COP/EP	Alive
8	73	M	ADC	Current	IV	4	0	No	Yes	Yes	N.E.	Gefitinib	13	AIP	Dead
9	65	F	ADC	Never	IV	2	3	No	No	No	N.E.	Gefitinib	38	HP	Dead
10	69	F	ADC	Never	IIIB	2	1	No	Yes	No	N.E.	Gefitinib	14	AIP	Dead
11	84	F	ADC	Ex	IIIB	4	0	No	Yes	No	Wild	Gefitinib	16	AIP	Dead
12	63	F	SCC	Never	IV	1	1	No	Yes	No	N.E.	Gefitinib	50	COP/EP	Dead
13	67	F	ADC	Never	Rec	0	1	No	No	No	Deletion	Gefitinib	48	HP	Alive
14	60	M	ADC	Current	IV	4	0	No	Yes	Yes	L858R	Gefitinib	17	HP	Dead
15	55	M	ADC	Ex	IV	3	4	No	No	No	L858R	Gefitinib	47	COP/EP	Dead
16	69	M	ADC	Current	IV	3	1	No	Yes	No	Wild	Erlotinib	14	AIP	Dead
17	56	M	SCC	Current	Rec	0	1	No	Yes	Yes	Wild	Erlotinib	21	COP/EP	Alive
18	59	M	ADC	Ex	Rec	1	2	No	Yes	No	Wild	Erlotinib	5	HP	Alive
19	66	M	ADC	Current	IIIB	0	0	No	No	No	L858R	Gefitinib	17	COP/EP	Alive
20	64	F	ADC	Never	Rec	1	1	No	No	No	L858R	Erlotinib	31	HP	Alive

Abbreviations: ADC, Adenocarcinoma; SCC, Squamous cell carcinoma; Rec, Recurrence after the surgery; CT, Chemotherapy; RT, Radiation therapy; N.E, Not evaluated; COP/EP, Cryptogenic organizing pneumonia/Eosinophilic pneumonia; HP, Hypersensitivity pneumonitis; DAD, Diffuse alveolar damage.

### Risk factors for developing EGFR-TKIs induced ILD

The results of univariate analyses on risk factors for EGFR-TKIs induced ILD are shown in Table 4. Univariate analyses revealed that only preexisting pulmonary fibrosis (odds ratio, 4.683; 95% CI, 1.741-12.042; *p *= 0.003) was a significant risk factor for the development of EGFR-TKIs induced ILD.

**Table 4:** Risk factors for EGFR-TKIs induced ILD at the start of EGFR-TKIs

Variables		Odds ratio	95% CI	*P*-value
Univariate analysis				
Age (years)	≥ 60/< 60	1.758	0.626-6.256	0.301
Gender	Male/Female	1.472	0.593-3.848	0.406
Histological type	Non-ADC/ADC	2.342	0.730-6.420	0.142
Smoking history	Never/Smoker	1.006	0.402-2.516	0.989
Performance status	≥ 2/0-1	0.942	0.360-2.341	0.899
No. of prior chemotherapy regimens	≥ 2/0-1	0.800	0.277-2.053	0.652
Prior thoracic radiotherapy	Yes/No	0.315	0.017-1.575	0.187
Preexisting pulmonary fibrosis	Yes/No	4.683	1.741-12.042	0.003*
Preexisting pulmonary emphysema	Yes/No	1.382	0.476-3.574	0.531
*EGFR *mutation	Wild type/*EGFR *mutant	1.667	0.497-5.594	0.400
Types of EGFR-TKI	Gefitinib/Erlotinib	2.043	0.562-5.948	0.253
Serum KL-6 level at baseline (U/ml)	≥ 500/< 500	2.096	0.679-7.116	0.199

*P < 0.05

Abbreviation: ADC, Adenocarcinoma.

### Serum levels of KL-6 in patients who developed EGFR-TKIs induced ILD

**Figure 3:**
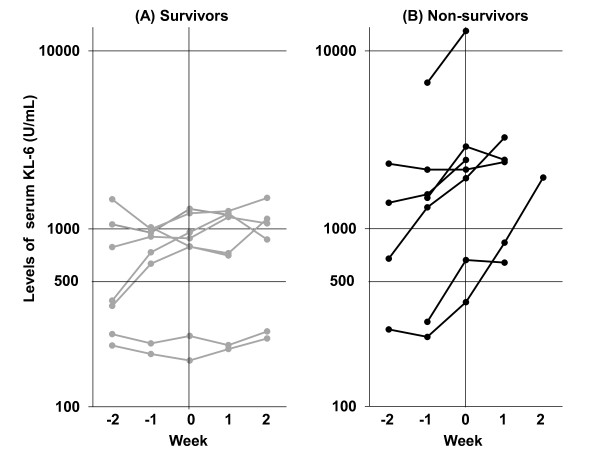
**Kinetics of serum KL-6 levels in (A) 8 survivors and (B) 7 non-survivors who developed EGFR-TKIs induced ILD**. Week 0 is designated as the week when EGFR-TKIs induced ILD was diagnosed. Before and after the onset of EGFR-TKIs induced ILD, the serum levels of KL-6 showed a trend not to change in the survivors but to increase in the non-survivors.

**Figure 4:**
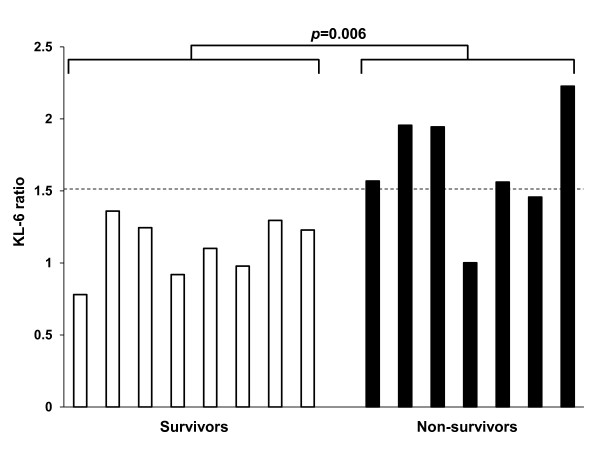
**The ratios of the serum levels of KL-6 at the onset of EGFR-TKIs induced ILD to those at baseline in 8 survivors and 7 non-survivors**. Open and solid bars represent survivors and non-survivors, respectively. There is a significant difference in these ratios between the survivors and non-survivors (*p *= 0.006).

After the administration of the EGFR-TKIs, measurements of serum KL-6 levels at least once during and/or around 4 weeks were achieved in 15 out of 20 patients who developed EGFR-TKIs induced ILD and 198 out of 321 patients who did not. The ratios of serum KL-6 levels during or around 4 weeks after the start of EGFR-TKIs to those at baseline were 1.315 ± 0.120 for the former and 1.000 ± 0.036 for the latter, respectively (mean ± SEM). There was a significant statistical difference between these ratios (*p *= 0.004, Mann-Whitney U-test). Figure 3 shows the serum levels of KL-6 at the multiple time points before and after the onset of ILD in 8 survivors (Figure 3A) and 7 non-survivors (Figure 3B). The serum levels of KL-6 in 7 non-survivors but not in 8 survivors showed consistent trends to increase after the onset of EGFR-TKIs induced ILD. The absolute serum KL-6 levels at the onset as well as at baseline showed no difference between the 7 non-survivors and 8 survivors (Mann-Whitney U-test; *p *= 0.072 at onset, and *p *= 0.072 at baseline, respectively). To assess the changes in serum KL-6 level before and after the onset of ILD, the ratio of serum KL-6 level just after the onset of ILD to that at baseline was calculated in 15 of 20 patients who developed ILD. The differences in the ratios of serum KL-6 levels just after the onset of ILD from baseline were found to be statistically significant between the survivors and non-survivors (Mann-Whitney U-test; *p *= 0.006; Figure 4).

**Figure 5:**
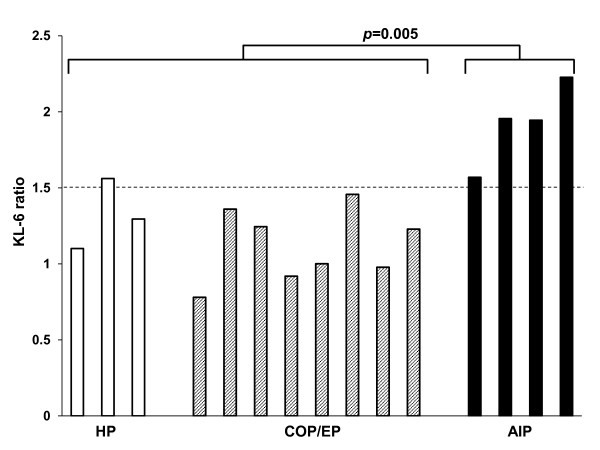
**The ratios of the serum levels of KL-6 at the onset of EGFR-TKI related ILD to those at baseline on the basis of the sub-classifications of EGFR-TKIs induced ILD**. Open, shaded, and solid bars represent hypersensitivity pneumonitis (HP) pattern, cryptogenic organizing pneumonia/eosinophilic pneumonia (COP/EP) pattern, and acute interstitial pneumonia (AIP) pattern, respectively. There is a significant difference in these ratios between AIP pattern and the other patterns (*p *= 0.005).

Then, we compared the circulating levels of KL-6 according to the patterns of EGFR-TKIs induced ILD subclassified by the manifestation on chest CT in 15 of 20 patients who developed EGFR-TKIs induced ILD. The absolute levels of circulating KL-6 at neither baseline nor the onset of ILD were found not to be statistically significant between the life-threatening pattern (AIP pattern) of 4 patients and the other patterns of 11 patients (Mann-Whitney U-test; *p *= 0.648 at onset, and *p *= 0.845 at baseline, respectively). When the ratio of serum KL-6 level at baseline to that at the onset of ILD was compared, this value was significantly higher in the patients with the life-threatening pattern (AIP pattern) than that in other patterns (Mann-Whitney U-test; *p *= 0.005; Figure 5). In addition, patients whose serum KL-6 levels rose more than 1.5 times higher than their baseline levels had a high chance of developing the AIP pattern.

## Discussion

In this large multi-institutional study, we investigated the incidence and risk factors for developing ILD in patients treated with EGFR-TKIs until 12 weeks after the start of EGFR-TKIs therapy. Univariate analyses revealed that preexisting pulmonary fibrosis at baseline was the only risk factor for EGFR-TKIs induced ILD. Although absolute serum KL-6 levels at neither baseline nor the onset of ILD could discriminate between life-threatening and non-life-threatening EGFR-TKIs induced ILDs, the ratio of serum KL-6 level at the occurrence of EGFR-TKIs induced ILD to that at baseline was found to quite precisely do so. These findings suggest the significance of serum KL-6 level for the detection of life threatening EGFR-TKIs induced ILD.

The development of molecular targeted agents has been a key factor in recent advances in cancer therapy, and some of these agents have been applied in clinical practice. EGFR-TKIs are one of the representative molecular target agents and, at first, were considered to be safe agents with mild side effects in comparison to cytotoxic agents. However, following the increase in usage of EGFR-TKIs in lung cancer therapy, a significantly higher incidence of life-threatening drug induced ILD in Japanese patients than that of patients in the rest of the world was reported [38,39]. In the present study, out of 341 NSCLC patients treated with EGFR-TKIs, 20 patients (5.9%) developed ILD and 9 patients (2.6%) died from ILD. The incidence and mortality of EGFR-TKIs induced ILD were relatively higher than those reported in previous studies from Japan [7-13,39]. This result might be due to the high incidence of preexisting pulmonary fibrosis in this study. In this study, the manifestations of chest CT scans in 20 patients who developed EGFR-TKIs induced ILD were classified as AIP pattern for 5 patients, COP/EP pattern for 9 patients and HP pattern for 6 patients. Interestingly, CIP pattern was not observed as was the case in a previous study [36]. All the patients who demonstrated the AIP pattern died, whereas the majority of patients with other patterns recovered from EGFR-TKIs induced ILD. In this study, the postmortem examination of three patients with AIP pattern revealed that DAD was the main cause of death and observations similar to ours have been reported previously [7,8]. In this study, univariate analysis revealed that preexisting pulmonary fibrosis was the only risk factor for developing EGFR-TKIs induced ILD. Although previous studies reported that male gender, smoking history and poor PS were also independent risk factors for developing EGFR-TKIs induced ILD [7-13,39], neither of them correlated with incidence or mortality of EGFR-TKIs induced ILD in the present study. This may be due to the small sample size and high incidence of preexisting pulmonary fibrosis in our studied patients.

Although a previous study from our laboratory reported that serum KL-6 levels at diagnosis increased only in the life-threatening types, such as the DAD and CIP patterns, of drug induced ILDs [27], absolute serum KL-6 levels at the onset of EGFR-TKIs induced ILD did not correlate with clinical outcomes in the present study. The immunohistochemical analysis of KL-6 using three postmortem autopsy specimens showed that KL-6 was expressed at tumor cells in the primary lesions as well as alveolar epithelial cells in the EGFR-TKIs induced ILDs (data not shown). Therefore, we speculate that the origin of serum KL-6 at the onset of EGFR-TKIs induced ILD might be associated with both NSCLCs and EGFR-TKIs induced ILDs. On the other hand, we found that the ratios of serum KL-6 levels just after the onset of ILD to those at baseline could quite precisely discriminate life-threatening ILD from non-life-threatening ILD, and correlate well with the disease progression. We can speculate that a drastic increase in serum KL-6 levels after the administration of EGFR-TKIs might be due to severe lung injury accompanied with both alveolar-capillary destruction and enhancement of alveolar-capillary permeability which allow KL-6 to leak into the circulation from the alveolar space [40]. Based on these observations, KL-6 can be regarded as a good serum biomarker to assess the severity of alveolar epithelium injury and the clinical outcome of EGFR-related ILD. Regarding the association between KL-6 and other serum biomarkers for ILD such as surfactant protein (SP)-A and SP-D in EGFR-TKIs induced ILD, we do not have data to discuss. Previous studies, which measured serum SP-A, SP-D, and KL-6 levels in 4 patients with EGFR-TKIs induced ILD, demonstrate that serum SP-A and SP-D levels increased in all studied patients whereas KL-6 levels only elevated in patients with life-threatening EGFR-TKIs induced ILD [8,41]. This observation is compatible with the findings of the present study.

In addition to its ability to detect patients who develop life-threatening ILD, the monitoring of serum KL-6 levels is also useful to predict survival and progressive disease in NSCLC patients treated with EGFR-TKIs [30]. As measurement of serum KL-6 level is more rapid, inexpensive, reproducible, and easier to perform than CT scans, its monitoring could be quite useful to assess the condition of NSCLC patients receiving EGFR-TKIs. The development of EGFR-TKIs induced ILD is reported to mostly occur within the first 4 weeks after the start of EGFR-TKIs [11]. In the present study, 5 cases developed ILD within the first 2 weeks (ranged from 5 to 14 days) after the start of EGF-TKIs. Therefore, based on the results of the present study, once a week monitoring of serum KL-6 levels in addition to chest radiography could be recommended for NSCLC patients receiving EGFR-TKIs particularly for the first 4 weeks after the start of treatment.

Although these promising results were obtained, we are aware that this study has a number of limitations. First, the number of EGFR-TKIs induced ILD patients included in the study was not sufficient for a valid statistical analysis. Second, this study was conducted in a retrospective manner. Therefore, the information on *EGFR *mutation statuses in cancer tissue was not obtained from all the studied patients. Furthermore, multiple measurements of serum KL-6 levels were not achieved in all patients who developed EGFR-TKIs induced ILD. Third, the enrolled NSCLC patients might be biased compared with general advanced NSCLC population. We believe that this was caused by our trend to use EGFR-TKIs for specific subgroups of NSCLC patients such as women, non-smokers, and patients with *EGFR *mutations. Finally, the studied patients were only Japanese. Considering ethnic differences in the efficacy of EGFR-TKIs treatment and/or the occurrence of adverse side effects related by EGFR-TKIs, we should carefully interpret the results when this monitoring system is applied to non-Japanese patients. A large and prospective study to measure serum KL-6 levels serially before and after EGFR-TKIs treatment, also including non-Japanese patients, will be required to evaluate the utility of monitoring KL-6 in EGFR-TKIs induced ILDs.

## Conclusions

Our results indicate that the change in serum KL-6 level from baseline should be useful biomarker for the diagnosis of life-threatening EGFR-TKIs induced ILD and for estimating its progress and severity. A risk-benefit analysis and patient selection should be considered as well as close monitoring of serum levels of KL-6, particularly if using EGFR-TKIs in patients with preexisting pulmonary fibrosis.

## List of Abbreviations

EGFR-TKI: epidermal growth factor receptor tyrosine kinase inhibitor; NSCLC: non-small cell lung cancer; ADC: adenocarcinoma; ILD: interstitial lung disease; KL-6: Krebs von den Lungen-6; IPF: idiopathic pulmonary fibrosis; AIP: acute interstitial pneumonia; CIP: chronic interstitial pneumonia; CT: computed tomography; FDG-PET: F-18 fluorodeoxyglucose positron emission tomography; MRI: magnetic resonance imaging; COP/EP: cryptogenic organizing pneumonia/eosinophilic pneumonia; HP: hypersensitivity pneumonitis; PNA-LNA PCR: peptide nucleic acid-locked nucleic acid polymerase chain reaction; ECLIA: electrochemiluminescence immunoassay.

## Competing interests

Nobuoki Kohno has a personal royalty of KL-6 from a Japanese pharmaceutical company, Eisai Co., Ltd. The remaining authors have no conflict of interest.

## Authors' contributions

SK performed part of the statistical analysis and drafted the manuscript. NH conceived the study, and participated in its design and coordination and helped to draft the manuscript. NI conceived the study, and participated in patient recruitment and helped to draft the manuscript. YH performed part of the statistical analysis and participated in creating the figures. KF, OF, TI, SM, HH and TY participated in the selection and collection of patient material. AY conceived the study, and participated in its design and coordination. NK conceived the study, and participated in its design and coordination and supervised the study. All authors read and approved the final manuscript.
